# Multimaterial Fibers Interfaced with ZnO for Photoelectrochemical Detection

**DOI:** 10.1002/smsc.202500468

**Published:** 2025-11-03

**Authors:** Supattra Somsri, Rayan Zaiter, Louis Rougier, Angéline Poulon‐Quintin, Catherine Boussard‐Plédel, Yann R. Leroux, Sébastien Chenu, Thierry Cardinal, Johann Troles, Gabriel Loget

**Affiliations:** ^1^ Univ. Bordeaux CNRS Bordeaux INP ISM‐UMR 5255 33607 Pessac France; ^2^ Univ. Bordeaux CNRS Bordeaux INP ICMCB UMR 5026 F‐33600 Pessac France; ^3^ Univ Rennes CNRS ISCR UMR 6226 F‐35000 Rennes France

**Keywords:** multimaterial fibers, optical sensor, photoanodes, photoelectrochemistry, ZnO

## Abstract

The development of miniaturized, remotely addressable sensing devices is crucial in a variety of fields, including healthcare, environmental monitoring, and security. This study introduces an optrode sensor comprising a multimaterial fiber composed of a phosphate glass cladding and a continuous Zn wire core, interfaced with a photoactive ZnO coating on its tip, deposited by anodization. It is shown that this optrode can promote photoelectrochemical reactions under illumination with UV light when immersed in an aqueous electrolyte. Proof‐of‐principle experiments demonstrate that these optrodes produce a glucose‐responsive photocurrent, opening the way to biomedical applications. This optical sensor shows promise, as it would ultimately allow the decoupling of input stimuli, i.e., potential and light excitation, over a long distance. Due to its advantages in terms of integration, detection speed, and ease of use, these ZnO/Zn/phosphate optrodes hold significant potential for remote analysis and implantable sensors.

## Graphical abstract

This study introduces an optrode sensor featuring a Zn wire core coated with phosphate glass and modified with a photoactive ZnO coating. It enables chemical reactions when exposed to ultraviolet light in a water‐based solution and can be used as a glucose sensor. This new family of sensors has strong potential for remote analysis and implantable analytical tools.

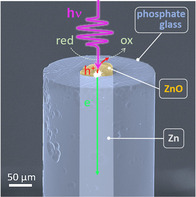

## 1 Introduction

Photoelectrochemical (PEC) analysis, which employs light as the excitation source and photocurrent as the sensing signal, is considered a sensitive and convenient method for the detection of various analytes.^[^ ^1^, ^2^, ^3^, ^4^, ^5^, ^6^, ^7^, ^8^, ^9^, ^10^, ^11^, ^12^ ^]^ In PEC sensors, the illumination of a semiconductor‐based photoelectrode, immersed in a solution, triggers the generation of a Faradaic photocurrent. The magnitude of which depends on the analyte concentration. When using a miniaturized photoelectrode, this technique presents several advantages, including rapid response, high sensitivity, compact size, low background signal, and minimal sample volume requirement.^[^ ^13^, ^14^, ^15^, ^16^ ^]^ Researchers are actively engaged in the development of various types PEC probes that facilitate rapid detection, ease of handling and cost‐effective analysis in biosensor applications.^[^ ^15^, ^16^, ^17^ ^]^ Nevertheless, the design of practically applicable PEC sensing architectures often encounters^[^ ^18^, ^19^, ^20^ ^]^ technical challenges for achieving the following requirements: robust collection of photogenerated carriers, optimal waveguiding, efficient interfacial charge transfer, and stability of the semiconductor in operation.^[^ ^21^, ^22^, ^23^ ^]^

**Figure 1:**
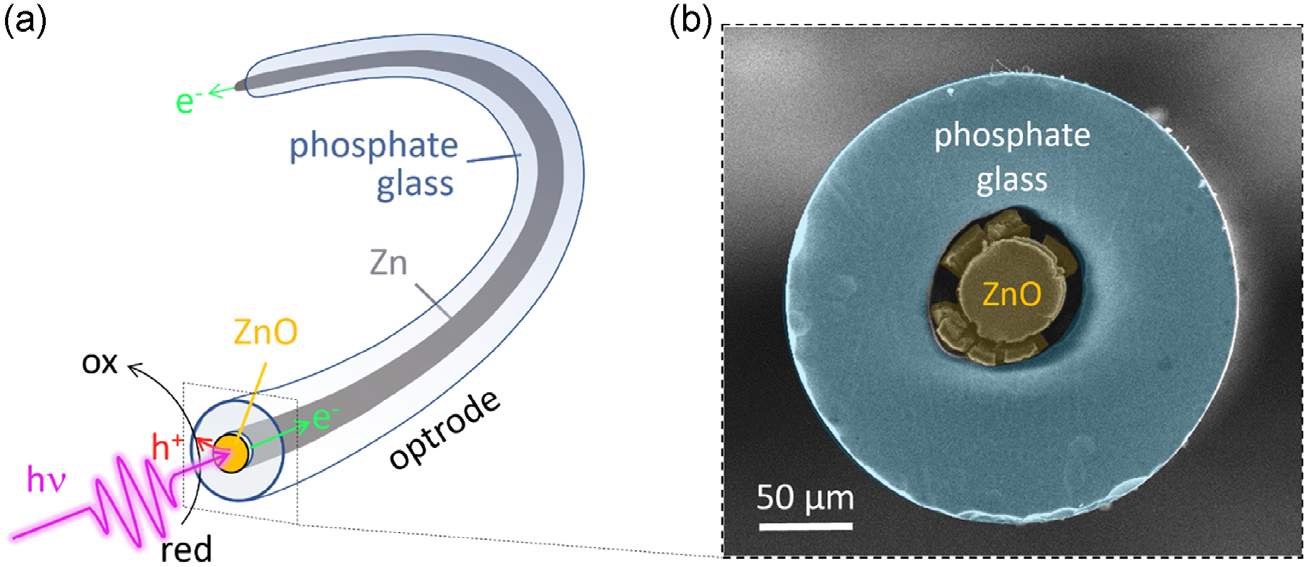
a) Scheme and b) colored SEM top view image of a ZnO/Zn/phosphate optrode.

Optical fibers have been extensively employed in the fields of photonics and optoelectronics^[^ ^24^ ^]^ as biosensors, including those utilizing chemiluminescence, electrochemiluminescence, surface plasmon resonance, and surface‐enhanced Raman spectroscopy.^[^ ^25^, ^26^, ^27^, ^28^, ^29^ ^]^ Multimaterial fibers combine several materials such as glasses, metals and/or polymers that are drawn and assembled to obtain hybrid fibers that can achieve several functionalities.^[^ ^30^, ^31^, ^32^ ^]^ The recent advances in optical fiber‐based biosensors have been considerable but only a few fiber‐based PEC sensors have been reported to date.^[^ ^6^, ^29^, ^33^, ^34^, ^35^, ^36^, ^37^ ^]^ In these works, the photoactive material requires an electronic connection external to the optical fiber.^[^ ^6^, ^29^, ^33^, ^34^, ^35^, ^36^, ^37^ ^]^ This can be problematic as it may induce undesired “dark” electrochemical reactions contributing to the overall photocurrent response. Furthermore, the techniques used to integrate semiconductors onto optical fibers are complex and involve multiple steps and leading to high system costs. Herein, we report the manufacturing and characterization of photoelectrochemically‐active optrodes by interfacing a photoactive ZnO coating onto a multimaterial fiber composed of a Zn “electrical wire” core embedded into a phosphate glass cladding, as shown in **Figure** **1**. This is, to the best of our knowledge the first example of a multimaterial fiber converted into a photoelectrode. It presents a straightforward method for synthesizing semiconductor ZnO through the anodization of Zn, which is more convenient than traditional thin film preparation techniques. Finally, proof‐of‐principle experiments reveal that these optrodes can be employed as glucose sensors.

## 2 Synthesis of the ZnO/Zn/phosphate Fibers

**Figure 2:**
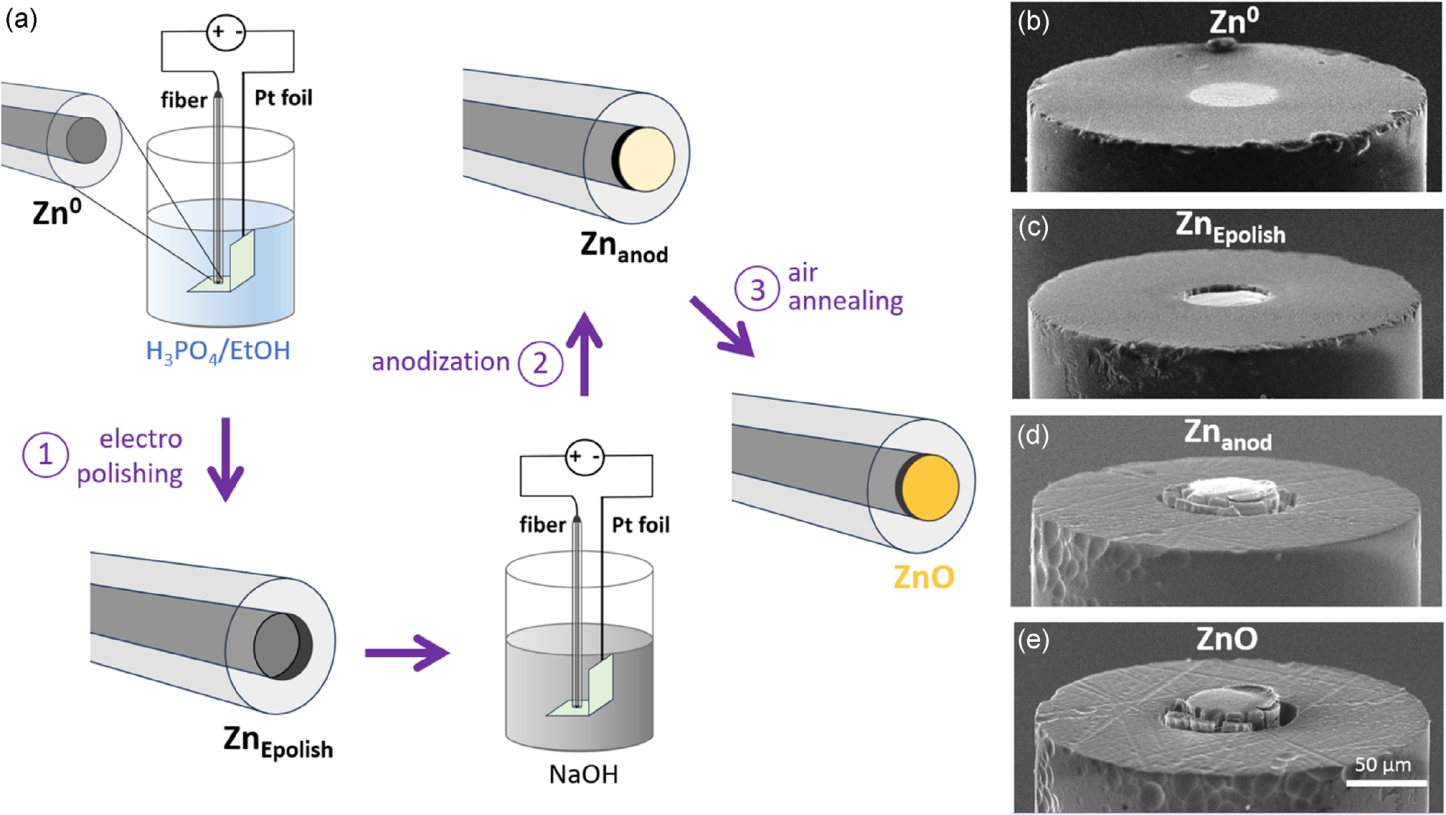
a) Scheme of the fabrication of a ZnO/Zn/phosphate optrode. b–e) SEM tilted side view of the extremity of multimaterial fibers showing b) Zn^0^, c) Zn_Epolish_, d) Zn_anod_, and e) ZnO.

The multimaterial fibers, comprising a Zn wire core electrically insulated by a phosphate glass cladding, were first manufactured (see the experimental section for detailed information). A cylindrical glass preform (diameter/height: 15/60 mm, composition: 40P_2_O_5_−20MgO−35Na_2_O−5Al_2_O_3_ mol%) was prepared by melting‐casting. A 2 mm‐large hole was mechanically drilled into this preform and Zn wires were inserted into the hole. Finally, the glass preform was drawn at 450 °C under Ar atmosphere using an optical fiber drawing tower. This resulted in the production of several meters of multimaterial fiber, denoted here as Zn/phosphate, with a diameter ranging between 180 to 250 μm depending on the drawing parameters. After fabrication, the Zn/phosphate fiber was cut into ≈8 cm‐long pieces that were mechanically polished on one side, as described in the experimental section. Then, the ZnO/Zn/phosphate optrodes, depicted in Figure 1, were prepared by the electrodeposition of a ZnO coating at one end of the Zn/phosphate fibers, as shown **Figure** **2**.

The deposition of ZnO onto the Zn^0^ extremity follows a three‐steps procedure inspired from Mika et al. (Figure 2a).^[^ ^38^, ^39^ ^]^ To ensure the reliability of the method, each step was controlled by scanning electron microscope (SEM). Figure 2b shows the Zn^0^ extremity of the initial Zn/phosphate fiber, which was first electropolished in a phosphoric acid (85%)/ethanol (1:2 v/v) mixture (10 V, 1 min). This step etched the Zn^0^ surface (see Figure S1, Supporting Information) by dissolving the outer part of the Zn^0^ piece, thereby producing a cavity (depth of ≈5 μm) at the tip of the fiber, as shown in Figure 2c. Then, Zn^0^ was anodized in a 1 m NaOH solution (2 V, 30 min), producing a ZnO coating directly onto the Zn surface recessed in the cavity. As indicated in the literature,^[^ ^40^ ^]^ the formation of ZnO during anodization process can be expressed with the following reactions
(1)

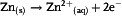



(2)

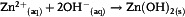



(3)

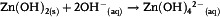



(4)

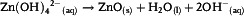




As shown in Figure 1b and Figure 2d, the ZnO coating extended over the cavity, and cracks were observed at the boundary between the phosphate cladding and the metal oxide core, creating a ZnO “corona”. Such a corona was systematically observed, as illustrated by a series of SEM pictures showing the ZnO of various optrodes, shown in Figure S2, Supporting Information. Both these phenomena are attributed to the volume expansion caused by the ZnO incorporation (molar volume of ZnO wurstite: 14.51 cm^3^ mol^−1^ against 9.16 cm^3^ mol^−1^ for Zn) and the associated mechanical constraints exerted on the metal oxide coating and the phosphate cladding during anodization. The occurrence of such strong mechanical constraints during anodization is also supported by the fact that the phosphate aperture diameter increased during anodization (by ≈30%, as shown in Figure S3, Supporting Information), which may have been favored by the heating of the glass via Joule effect during anodization. In addition, it was found that the thickness of the ZnO film deposited on a fiber was greater than the one deposited on a similarly treated Zn foil (≈7 vs. 3 μm, see Figure S4, Supporting Information). Finally, the multimaterial fiber was annealed in air at 400 °C to improve the semiconductor crystallinity. Top and side‐view SEM pictures of the photoactive extremity of typical ZnO/Zn/phosphate optrodes are shown in Figure 1b and Figure 2e, respectively.

**Figure 3:**
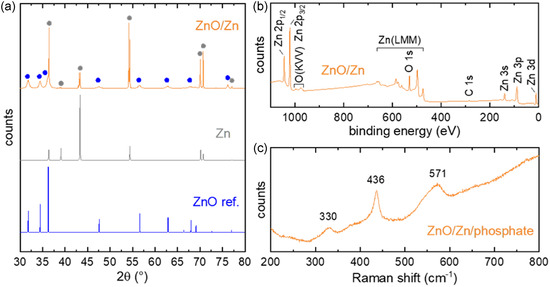
a) Theoretical (blue) and experimentally measured (gray and orange) XRD patterns for ZnO (COD: 9004178, (blue)), Zn foil (gray), and ZnO/Zn foil (orange). b) XPS survey spectrum of ZnO/Zn foil. c) Raman spectrum recorded on a ZnO/Zn/phosphate optrode.

X‐ray diffraction (XRD) and X‐ray photoelectron spectroscopy (XPS) were acquired on Zn foils that were processed exactly as the fibers. The ZnO/Zn experimental XRD pattern (orange in **Figure** **3**a), compared with that of a Zn foil (gray) and a reference ZnO diffractogram (blue XRD pattern, COD: 9 004 178), indicates, after annealing, the presence of a well crystallized wurtzite ZnO (blue dots in Figure 3a) onto the Zn substrate (gray dots in Figure 3a). The XPS survey spectrum of ZnO/Zn, shown in Figure 3b, presents the expected Zn and O peaks^[^ ^41^, ^42^ ^]^ and the absence of contaminants (except for adventitious carbon, as indicated by the C 1s peak at 285 eV). The Zn/O ratio was analyzed as a function of depth, as shown in the profile of Figure S5, Supporting Information. The stoichiometry varied from 0.8 to 1.1 while going deeper inside the coating, which indicates O vacancies inside the deposited ZnO. A Raman spectroscopy analysis was performed on a ZnO/Zn/phosphate optrode, as described in the experimental section. Figure 3c shows a typical Raman spectrum, which presents peaks at 330, 436, and 571 cm^−1^. The prominent band at 436 cm^−1^ corresponds to the E_2_(high) mode, characteristic of the wurtzite crystal structure,^[^ ^43^ ^]^ which confirms the crystalline quality of the coating. The band at 330 cm^−1^ is attributed to a second‐order multiphonon process, specifically a difference mode involving the E_2_(high) and E_2_(low) phonons.^[^ ^44^ ^]^ The band at 571 cm^−1^ is commonly associated with the presence of O vacancies in the lattice,^[^ ^43^ ^]^ in good agreement with the previously discussed XPS depth profile (Figure S5, Supporting Information). Raman mapping was performed over an 8 × 8 μm^2^ area of the ZnO coating onto the ZnO/Zn/phosphate optrode to evaluate the spatial distribution of the characteristic vibrational modes. The ratio of the intensity of the E_2_(high) mode at 436 cm^−^^1^ to the defect‐related band at 571 cm^−^^1^ is related to the ZnO stoichiometry. As shown in Figure S6a, Supporting Information the Raman map demonstrates a uniform distribution across the scanned zone, indicating high spatial homogeneity in the structural and defect characteristics within the probed ZnO volume. To investigate the effect of anodization on the electronic structure of the ZnO coating, XRD, SEM, and photoluminescence (PL) measurements performed on Zn foils after anodization + annealing and after a direct annealing of the Zn foil were compared. As shown in Figure S7a, Supporting Information ZnO obtained from direct annealing exhibits a much lower signal than the one prepared by anodization + annealing. This is due to the formation of a much thinner ZnO layer on Zn foil (0.3 vs. 7 μm), as confirmed by SEM in Figure S7b, Supporting Information. Moreover, as shown in Figure S8a, Supporting Information the two treatments result in significantly different PL emission profiles. The ZnO sample obtained by direct annealing exhibits a strong near‐band‐edge (NBE) emission centered around 380 nm, corresponding to direct radiative recombination of electrons in the conduction band with holes in the valence band, and a green emission band centered at 515 nm, related to the presence of oxygen vacancies.^[^ ^45^ ^]^ Meanwhile, the ZnO sample obtained by anodization followed by annealing shows a markedly quenched NBE emission and an enhanced broad visible emission with a maximum at 650 nm, indicating a higher density of intra‐bandgap states associated with intrinsic defects such as single and doubly ionized oxygen vacancies.^[^ ^46^ ^]^ Figure S8b, Supporting Information further highlights the differences in the excitation behavior. PL excitation spectra were recorded at fixed emission wavelengths of 515 and 648 nm, corresponding to green and red defect‐related emissions, respectively. The anodized sample shows an extended excitation response into sub‐bandgap photon energies, which suggests the presence of deep‐level states within the bandgap that facilitate radiative transitions even below the ZnO bandgap (approximately between 390 to 430 nm). Overall, the PL results confirm that anodization introduces or stabilizes additional defect states in the ZnO structure. To sum up, the SEM of Figure 2b–e and the characterization results of Figure 3a–c, obtained on ZnO/Zn foils and ZnO/Zn/phosphate optrodes, confirmed the successful integration of crystalline ZnO onto the multimaterial fibers. Next, we study the PEC properties of these miniaturized devices.

**Figure 4:**
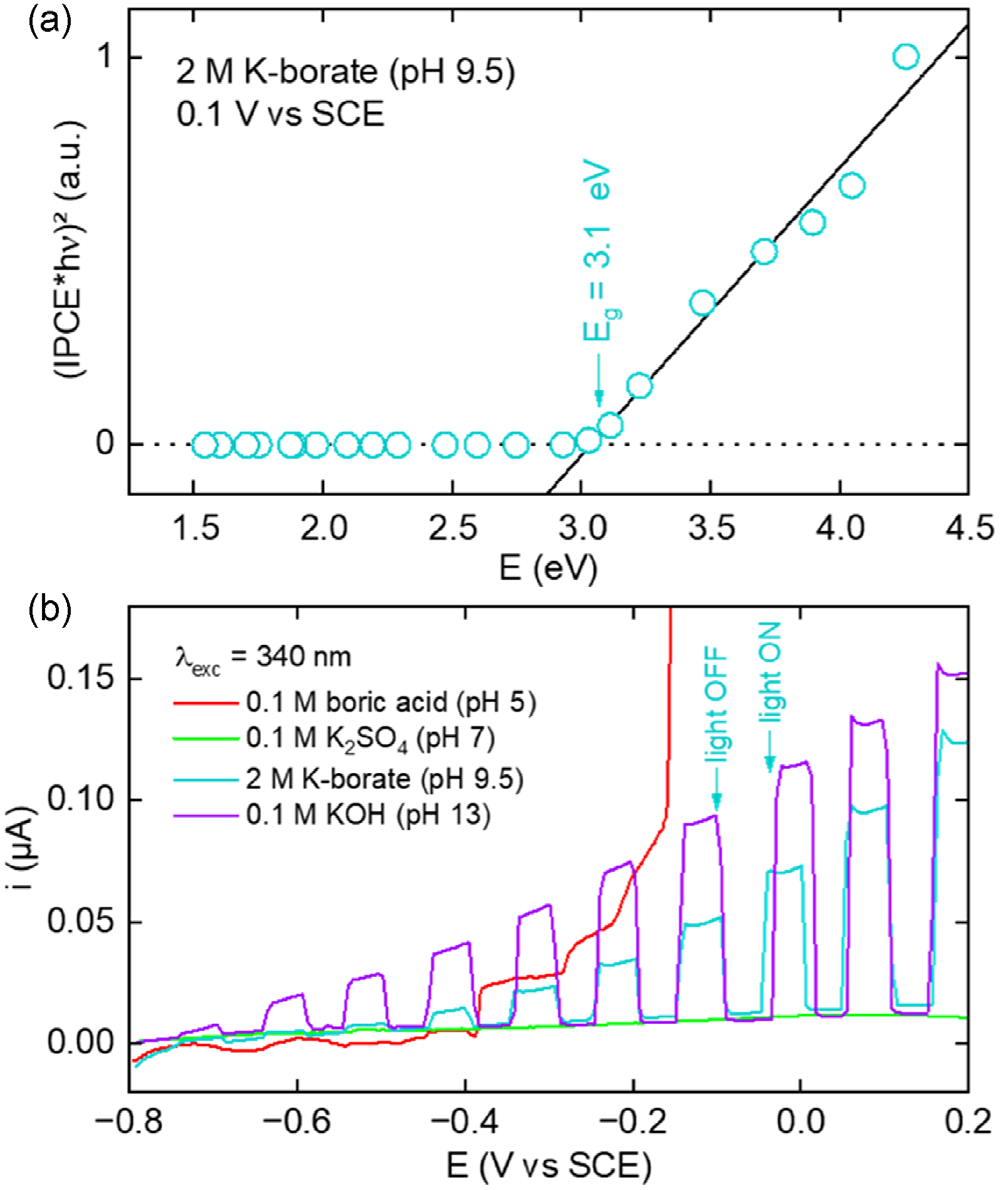
a) Tauc plot showing the ZnO photoelectrochemical bandgap, measured at 0.1 V in 2 M K‐borate. b) LSVs recorded with ZnO/Zn/phosphate optrodes under intermittent illumination with *λ*_exc_ = 340 nm in different electrolytes: 0.1 m boric acid (red curve), 0.1 m potassium sulfate (green curve), 2 m K‐borate (blue line), and 0.1 m KOH (purple line). Scan rate = 10 mV s^−1^.

First, the PEC response of ZnO/Zn/phosphate optrodes manufactured by the process shown in Figure 2 was compared to that of optrodes made by direct annealing of Zn/phosphate optrodes (400 °C in air). The linear sweep voltammograms (LSVs) of Figure S7c, Supporting Information reveal that the optrodes obtained via direct annealing presented a higher dark current at low overpotential compared to that resulting from ZnO from anodization + annealing. This indicates a poor shielding of the Zn wire and is attributed to the too thin ZnO layer (≈0.3 μm). Conversely, anodization generates a thicker coating (≈7 μm), which decreases the dark current. To determine the bandgap (*E*_g_) of the deposited ZnO, photocurrent spectroscopy was performed with a ZnO/Zn foil treated as the fiber. The resulting photocurrent spectrum, obtained at 0.1 V (all potentials in this article are referred vs. the saturated calomel electrode (saturated KCl), (SCE)) in a 2 m potassium borate (K‐borate) solution (pH = 9.5), is presented in the form of a Tauc plot in **Figure** **4**a. This graph indicates a *E*_g_ value of 3.1 eV, in good agreement with the value of 3.1–3.4 eV commonly reported in the literature for ZnO.^[^ ^47^, ^48^, ^49^, ^50^, ^51^ ^]^ Next, to assess the stability of the optrodes in operating conditions, we studied the photoresponse of the ZnO/Zn/phosphate optrodes in various electrolytes in the PEC cell shown in Figure S9, Supporting Information. Figure 4b presents LSVs recorded under intermittent 340 nm (3.6 eV) illumination in four electrolytes with pH ranging from 5 to 13. The red LSV, measured at pH = 5, exhibits a low light response and a sharp oxidation current above –0.4 V. This behavior reveals the instability of ZnO/Zn and indicates that ZnO and Zn^0^ dissolution to Zn^2+^ occurs in these conditions, in good agreement with the behavior expected from the Pourbaix diagrams of Zn.^[^ ^52^ ^]^ The green LSV, recorded in K_2_SO_4_ (pH = 7) presents a negligible current (<15 nA) over the whole potential range investigated. This reveals the formation of a passive layer, likely an insulating oxide/hydroxide film,^[^ ^53^, ^54^ ^]^ which inhibits the overall electrochemical activity of the system, in good agreement with the literature.^[^ ^55^, ^56^ ^]^ Conversely, the alkaline electrolytes (K‐borate, pH 9.5 and KOH, pH 13) promoted a satisfying PEC response, as revealed by a low dark current (<15 nA) and a high and fast photocurrent response. Despite the higher photocurrent obtained in KOH, the photocurrent in KOH drops by ≈74% after chronoamperometry (CA) under illumination for 1 h. Instead, long‐term stability evaluation revealed a better stability in the K‐borate electrolyte with a photocurrent drop of only 36% (Figure S10, Supporting Information) and will be used in the next experiments.

**Figure 5:**
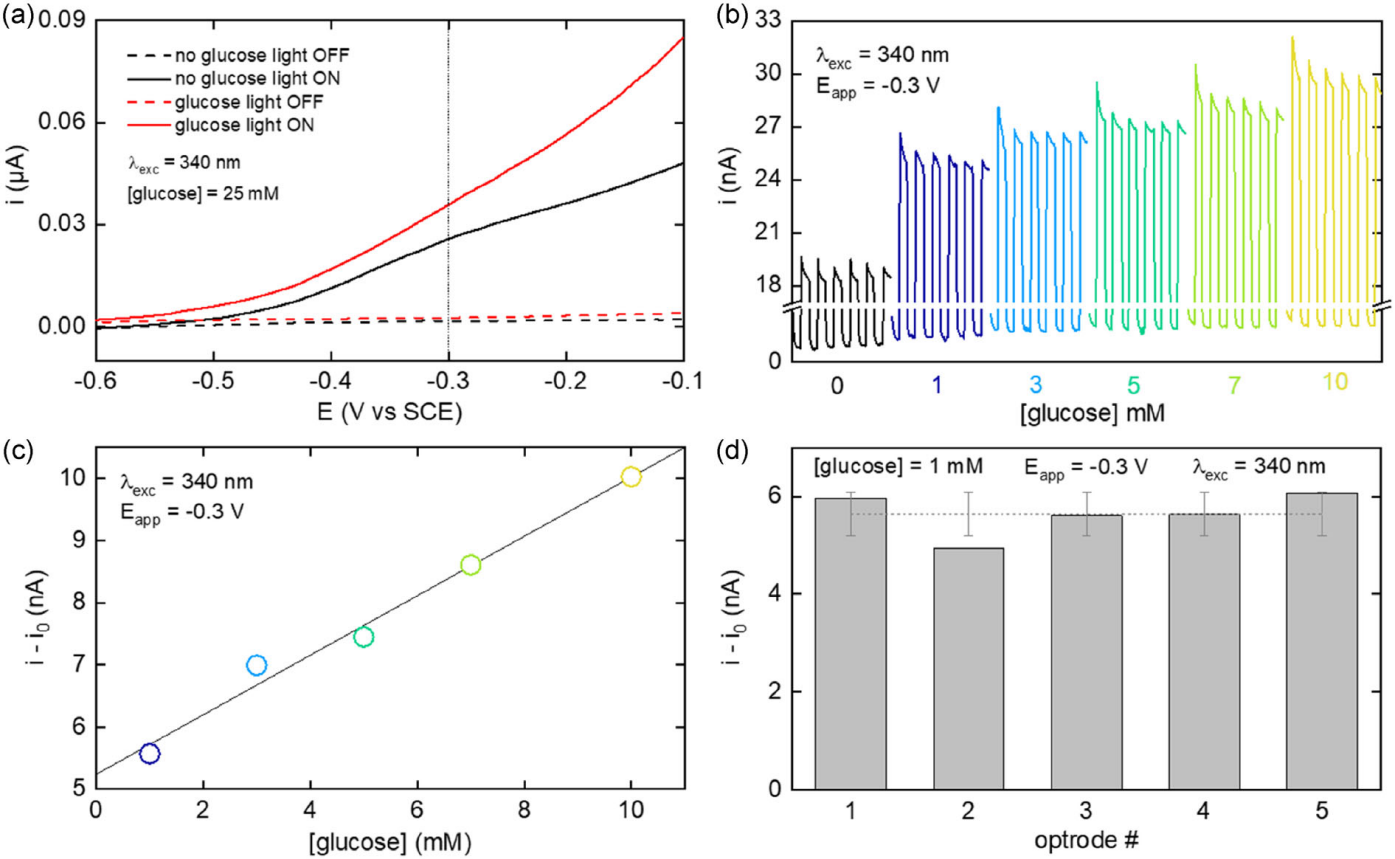
a) LSVs recorded with a ZnO/Zn/phosphate optrode in the dark (dashed curves) and under illumination (full line curves) in the absence (black curves) and in the presence (red curves) of 25 mM glucose. The vertical dotted line indicates the potential employed in the experiments of panels b–d). Scan rate = 10 mV s^−1^. b) Plot showing CAs recorded at −0.3 V under intermittent illumination (illumination frequency = 33 mHz) with different concentrations of glucose. c) Plot showing the evolution of the photocurrent variation (*i*–*i*_0_) as a function of glucose concentration, discs: experimental datapoints, line: linear fit (R^2^ = 0.986). d) Graph showing the photocurrent variation (*i*–*i*_0_) recorded at −0.3 V with five independently prepared ZnO/Zn/phosphate optrodes for a concentration of glucose of 1 mM. Colored bars: experimental data, dotted line: average value, the error bars equal 2 standard deviations. Electrolyte: 2 m K‐borate, illumination: 340 nm.

Finally, the ZnO/Zn/phosphate optrodes were tested for PEC glucose sensing. **Figure** **5**a shows LSVs obtained in the absence (black curves) and in the presence of 25 mM glucose (red curves). In agreement with the results of Figure 4b, the anodes produced negligible current in the dark but a higher photocurrent under 340 nm illumination. The comparison between the red and the black curves indicates that the presence of glucose promotes higher photocurrent, suggesting that glucose can be efficiently oxidized on ZnO. The influence of glucose concentration (up to 10 mM) was tested at a potential of –0.3 V. Figure 5b presents the resulting chronoamperograms (CAs), recorded under intermittent illumination. The photocurrent variation, corresponding to photocurrent recorded in the presence of glucose (*i*) minus that recorded in the absence of glucose (*i*_0_), is plotted in Figure 5c as a function of the glucose concentration. This calibration curve is linear in the studied concentration range with a sensitivity of 0.48 nA mM^−1^ (or 18.74 μA cm^−2^ mM^−1^, taking into account the projected geometrical surface area of the Zn disk) and a *R* ^2^ of 0.986. Compared to prior publications on ZnO PEC glucose sensors (as shown in Table S1, Supporting Information), this study demonstrates a significantly broader linear response range (1–10 mM) and increased sensitivity. In addition, the reproducibility of the optrode preparation and the glucose sensing was also studied. The photocurrent variation, recorded for a glucose concentration of 1 mM with five different ZnO/Zn/phosphate optrodes was plotted in Figure 5d. This plot reveals an average output signal of 5.60 ± 0.45 nA. In addition, this graph shows that the differences in morphology and active surface area between different optrodes do not significantly affect the overall output signal. Finally, the photocurrent response, assessed on a planar ZnO/Zn foil in the presence of various potential interferent (Figure S11, Supporting Information),^[^ ^57^ ^]^ indicates a good compatibility of the deposited ZnO for glucose measurements in complex media. To sum up, these proof‐of‐concept experiments reveal the feasibility of using the ZnO/Zn/phosphate optrodes for glucose sensing and the satisfying reproducibility of our method.

## 3 Conclusion

In summary, we have developed an optrode sensor composed of a multimaterial fiber containing a phosphate glass outer layer and a Zn wire core, modified at one extremity by a photoactive ZnO layer. The photoactive ZnO was deposited on Zn wire optrode sensor by anodization, which allowed, for the first time, to convert a multimaterial fiber into a photoelectrode. With respect to conventional photoelectrodes studied so far, the approach proposed here can provide several functional features, which are: 1) extreme length of the PEC device, which can that be made several meters long (Figure S12a, Supporting Information). sensor flexibility (see Figure S12b, Supporting Information), 2) noninvasiveness, and 3) embedded electrical contact. Experiments showed that this innovative ZnO/Zn/phosphate optrode is capable of inducing PEC reactions upon exposure to UV light in an aqueous solution. Moreover, experimental results demonstrate that this optrode reliably detects glucose within a concentration range of 1–10 mM, which holds potential utility in biomedical applications. This model system paves the way for the manufacturing of multimaterial optrode systems offering a high degree of flexibility, efficient light direction, minimal light loss, long‐distance information transmission, and reduced sample requirements. These attributes render them promising for flexible sensors, remote detection, and implantable monitoring devices. These results open the door to the preparation of a new generation of sensing devices where flexibility, light guiding, and remote accessibility will outperform that obtained by thin film fabrication methods. In future research, we intend to further investigate the selectivity of our sensor toward glucose in the presence of interfering substances, as well as its long‐term performance. Additionally, we aim to extend this concept to other semiconductor materials, such as BiVO_4_ and Fe_2_O_3_, to enhance sensor performance and its response to visible spectra. Furthermore, we aspire to develop this device into a compact microdevice that integrates working, counter, and reference electrodes within a single fiber.

## 4 Experimental Section

4.1

4.1.1

##### Synthesis of ZnO on Zn Foils

A zinc foil (99.98%, 0.25 mm thick, Thermo Scientific) was cut into small pieces with dimensions of 1.0 × 2.0 cm^2^ and cleaned by ultrasonication in ethanol and acetone. The electrochemically area of Zn foil was limited by hydrophobic tape to 1.0 × 1.0 cm^−^^2^. The synthesis procedure of ZnO was adapted from literature.^[^ ^38^, ^39^ ^]^ First, the Zn foils were electrochemically polished using a two‐electrode setup in a mixture of 85% phosphoric acid^[^ ^38^, ^39^ ^]^ and ethanol 1:2 v/v at a potential of 10 V for 1 min at room temperature. After cleaning with ultrapure water several times, Zn_Epolish_ was obtained. Zn_Epolish_ was anodized in 1 M NaOH electrolyte using a similar setup at a constant potential of 2 V for 30 min at room temperature to obtain Zn_anod_. The final ZnO was obtained from annealing process under air at 400 °C for 2 h with a heating rate of 10 °C min^−1^. Electropolishing and anodization were carried out in a two‐electrode system using the Zn foil as an anode and a Pt foil as a cathode. The ZnO on Zn foil was used for XRD, XPS, PL, and photocurrent spectroscopy characterizations.

##### Synthesis of Zn/phosphate Multimaterial Fibers

A phosphate glass preform of the composition 40P_2_O_5_−20MgO−35Na_2_O−5Al_2_O_3_ (mol%) was prepared by the conventional melting‐casting technique. The precursors: (NaPO_3_)_6_ (Thermofisher), H_3_PO_4_ (Roth, ≥85%), MgO (Fox chemicals, 99.99%) and Al_2_O_3_ (Fox chemicals, 99.998%) were weighed in adequate molar rations, mixed and placed in a Teflon beaker at 270 °C in a sand bath overnight to form a homogeneous mixture. The resulting mixture was ground into a fine powder, transferred to a platinum crucible and heated up to 1200 °C for 2 h. The melt was then cast into a cylindrical graphite mold with an inner diameter of 15 mm. Once cast, the 6 cm‐long glass preform was annealed at the T_g_ (440 °C) for 12 h then slowly cooled down to room temperature to reduce internal stress. A hole was mechanically drilled into the preform using a 2 mm‐diameter drill to a depth of 3 mm, and two 1 mm‐diameter zinc wires were inserted into the hole. Finally, the glass preform was drawn homothetically using an optical fiber drawing tower. The preform was thermally heated at a rate of 10 °C min^−1^ under Ar flow (3 L min^−1^) up to 450 °C to initiate the stretching process. It was then fed slowly into the annular furnace at a rate of 1 mm min^−1^. The drawing parameters were continuously monitored, resulting in the production of several meters of Zn/phosphate multimaterial fibers with diameter of 180–250 μm.

##### Synthesis of the ZnO/Zn/phosphate Optrodes

The Zn/phosphate multimaterial fibers were cut into length of 8 cm and one tip of the fiber was mechanically polished. Then another tip of the fiber was connected with an electrical wire using conductive silver paste and then covered with epoxy resin. After that, the Zn/phosphate multimaterial fibers were electropolished in a mixture of 85% phosphoric acid and ethanol 1:2 v/v at a potential of 10 V for 1 min at room temperature (same condition as for the Zn foils). Zn_anod_/Zn/phosphate was also prepared by the same anodization method as that employed with the Zn foil. Finally, ZnO/Zn/phosphate optrodes were prepared by annealing the Zn_anod_/Zn/phosphate fibers under air at 400 °C respectively for 2 h with a heating rate of 10 °C min^−1^. ZnO/Zn/phosphate optrodes were employed for Raman, SEM, and photoelectroanalytical analyses.

##### Characterizations

The morphology of ZnO and Zn surfaces was analyzed using a Lyra 3GM (Tescan) SEM. The crystallinity of samples was verified using a X‐ray diffractometer with monochromatric Cu K_α1_ radiation (*λ* = 1.5418 Å).^[^ ^58^ ^]^ XPS was performed using a K‐Alpha apparatus (ThermoFisher 391 Scientific) with a monochromatized Al‐Kα source (1486.7 eV). The electron binding energy (BE) scale was calibrated relatively to C 1s maximum at BE 285 eV. Raman spectra were collected using a confocal micro‐Raman spectrometer (LabRAM HR Evolution, Horiba Jobin Yvon), equipped with a Synapse charge coupled device detector using a 532 nm laser diode as the excitation source. The incident laser beam was focused onto the sample through a 100x objective (NA = 0.80, Olympus). Scattered light was dispersed by a holographic grating with 1800 grooves/mm. PL spectra were measured at room temperature using a Horiba Jobin Yvon Fluorolog 3, equipped with a Xenon lamp, a double excitation monochromator, an iHR320 emission monochromator and a Hamamatsu R298 photomultiplier tube detector. PEC measurements were conducted in homemade cell with a quartz window in a three‐electrode setup, where the ZnO on Zn fiber was used as working electrode, Pt wire as counter electrode, a SCE as a reference electrode and a LED light (Thorlabs) at 340 nm (power density = 7.33 mW cm^−2^) as a light source. The generated photocurrents were recorded using a SP‐150 (BioLogic) potentiostat.

## Supporting Information

Supporting Information is available from the Wiley Online Library or from the author.

## Conflict of Interest

The authors declare no conflict of interest.

## Supporting information

Supplementary Material

## Data Availability

The data that support the findings of this study are available from the corresponding author upon reasonable request.
