# Supplementary material for: Multimaterial Fibers Interfaced with ZnO for Photoelectrochemical Detection
Source: Small Sci. 2025 Nov 3;5(12):e202500468. doi: 10.1002/smsc.202500468 (PMC12697863; doi:10.1002/smsc.202500468)
Supplement: Supplementary file 1 — Supplementary Material [file SMSC-5-e202500468-s001.pdf]

Supporting Information

Supporting Information is available from the Wiley Online Library or from the author.

This study introduces an optrode sensor featuring a Zn wire core coated with phosphate glass and modified with a photoactive ZnO coating. The optrode enables chemical reactions when exposed to UV light in a water-based solution and can be used as a glucose sensor. This new family of sensors has a strong potential for remote analysis and implantable analytical tools.

Multimaterial Fibers Interfaced with ZnO for Photoelectrochemical Detection

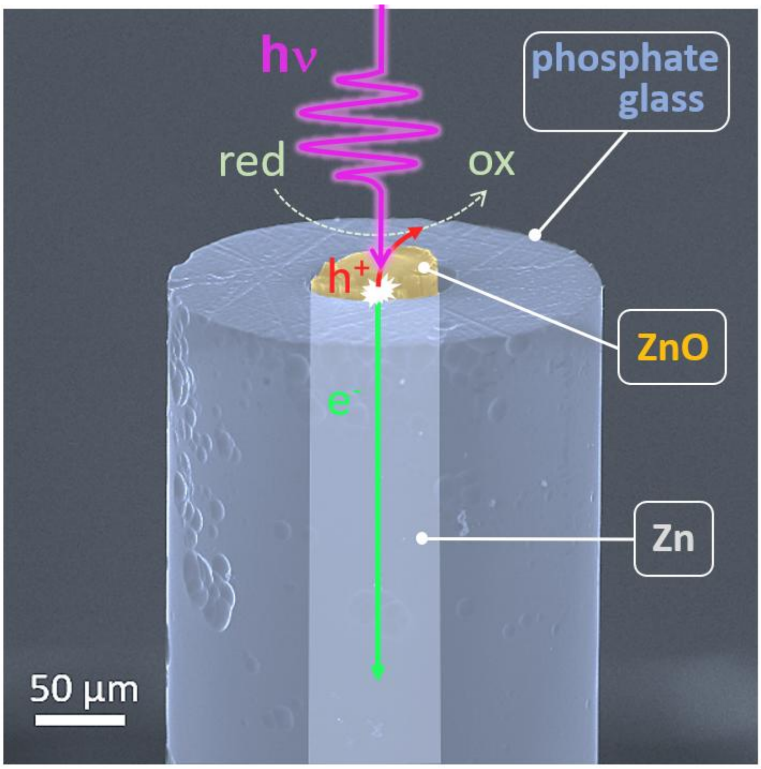

Supporting Information

Multimaterial Fibers Interfaced with ZnO for Photoelectrochemical Detection

*Supattra Somsri, Rayan Zaiter, Louis Rougier, Angéline Poulon-Quintin, Catherine Boussard-Plédel, Yann R. Leroux, Sébastien Chenu, Thierry Cardinal, Johann Troles, Gabriel Loget**

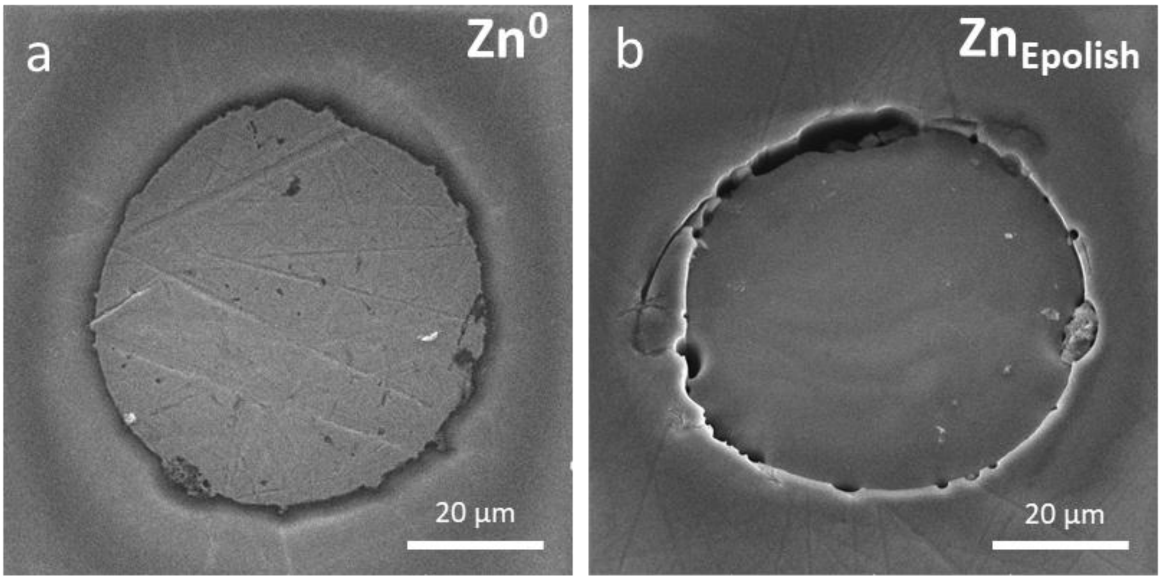

Figure S1. SEM top view images showing (a) Zn^0 and (b) $Zn_{Epolish}$ at the extremity of a multimaterial fiber.

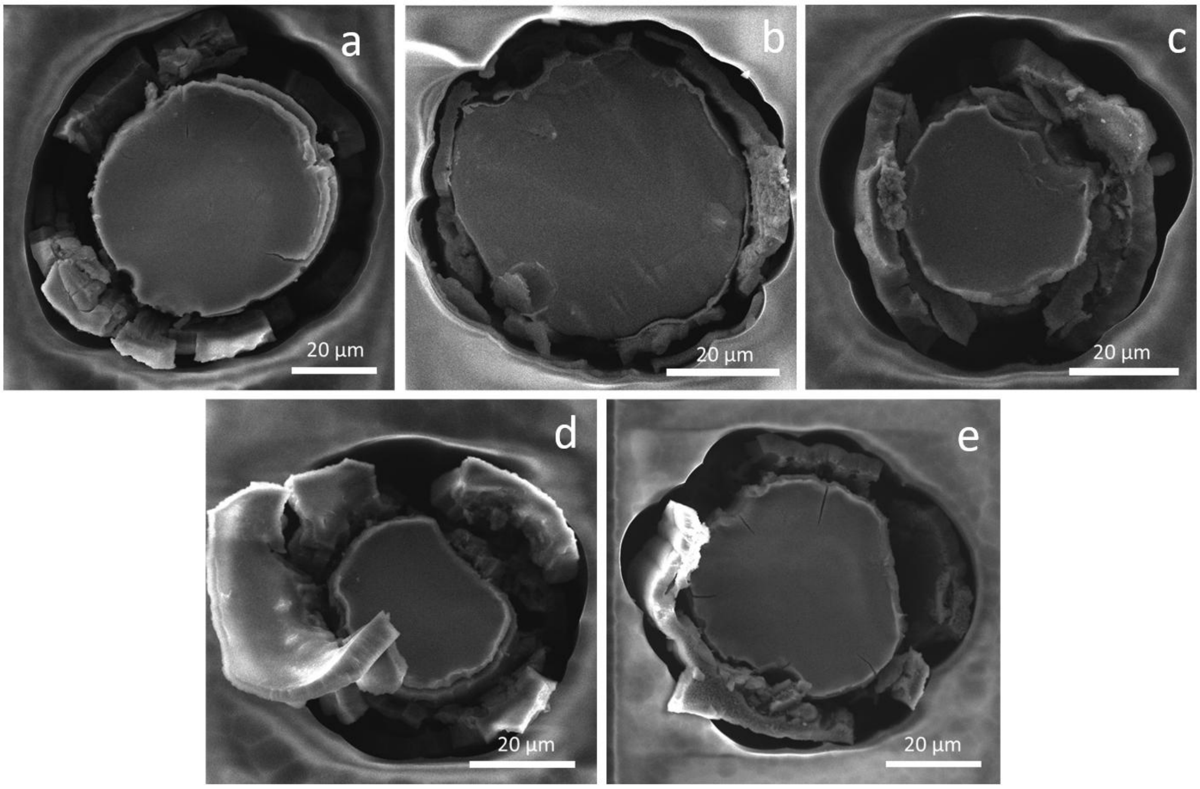

Figure S2. SEM top view images of several ZnO/Zn/phosphate optrodes.

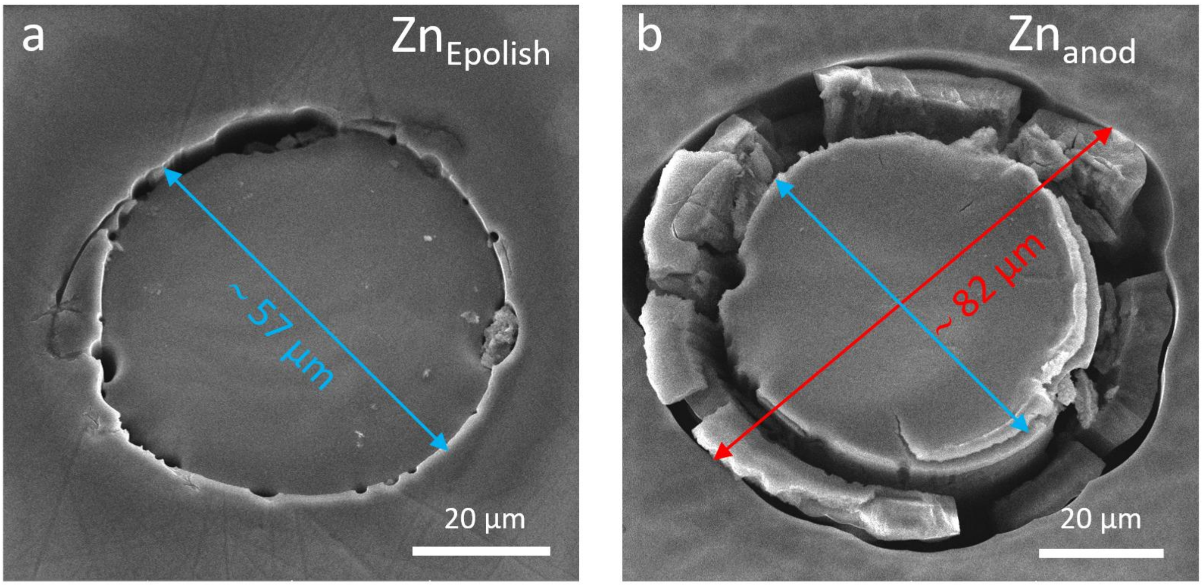

Figure S3. SEM images of (a) $Zn_{Epolish}$ and (b) Zn_{anod}, the arrows indicate the diameter of the glass aperture.

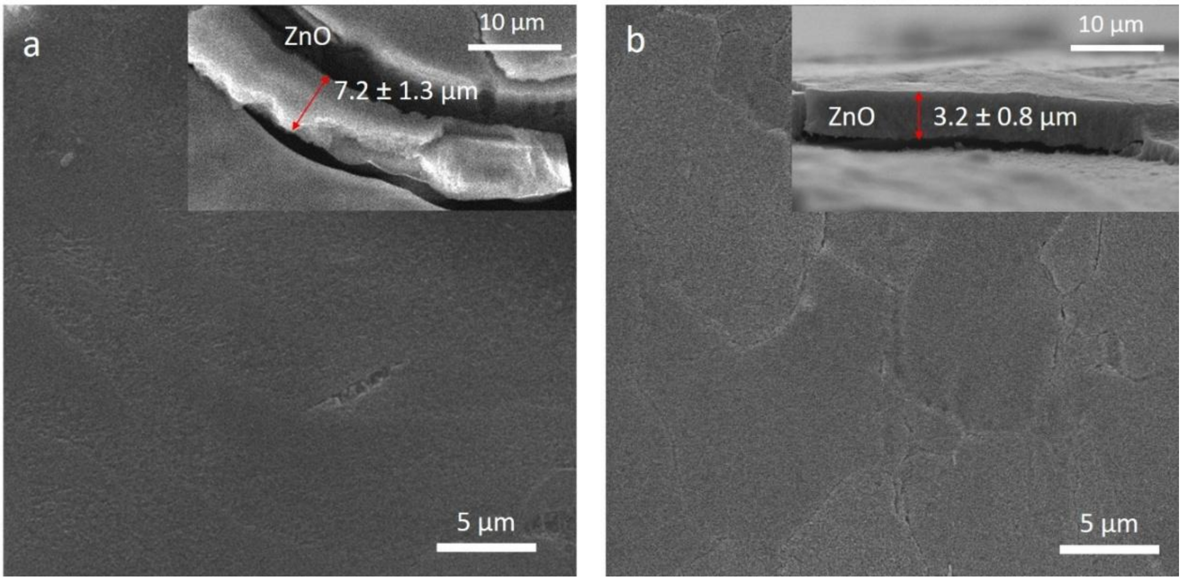

Figure S4. SEM images of (a) ZnO deposited Zn fiber and (b) ZnO on a Zn foil, both with the same method (as in Figure 2). The insets present the thickness of the ZnO film.

a

Etch Time (s)	Atomic % (%)			Zn/O atomic ratio
	C 1s	O 1s	Zn $2p_{3/2}$	
0	33.88	37.27	28.86	0.77
10	21.12	39.99	38.89	0.97
20	14.56	42.18	43.26	1.03
60	8.92	43.80	47.28	1.08

b

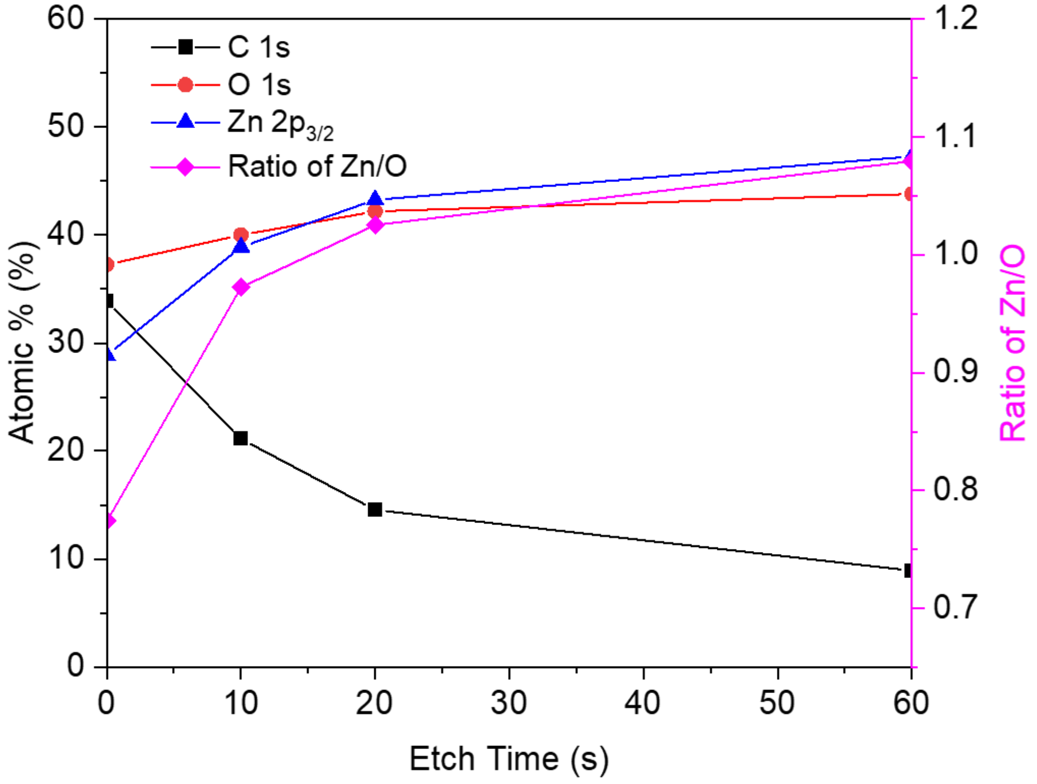

Figure S5. (a) Table and (b) figure showing the relative surface atomic percentages of C, O, and Zn on the ZnO (deposited on Zn foil) from XPS analysis as a function of etching time using plasma. The atomic ratio of Zn/O is also shown (pink curve in panel (b)).

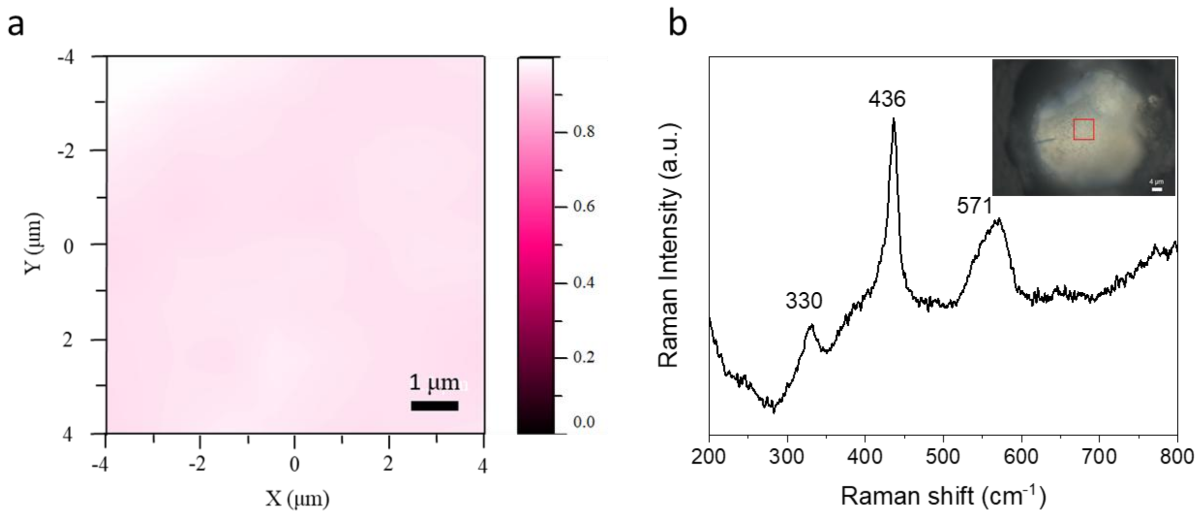

Figure S6. (a) Raman mapping, measured on a ZnO/Zn/phosphate optrode, showing the integrated peak area ratio of the 571 cm^{-1} and 436 cm^{-1} bands, (b) Single-point Raman spectrum from the mapped ZnO/Zn/phosphate optrode surface. The inset in (b) is an optical image that indicates the 8×8 μm^2 area investigated on the optrode.

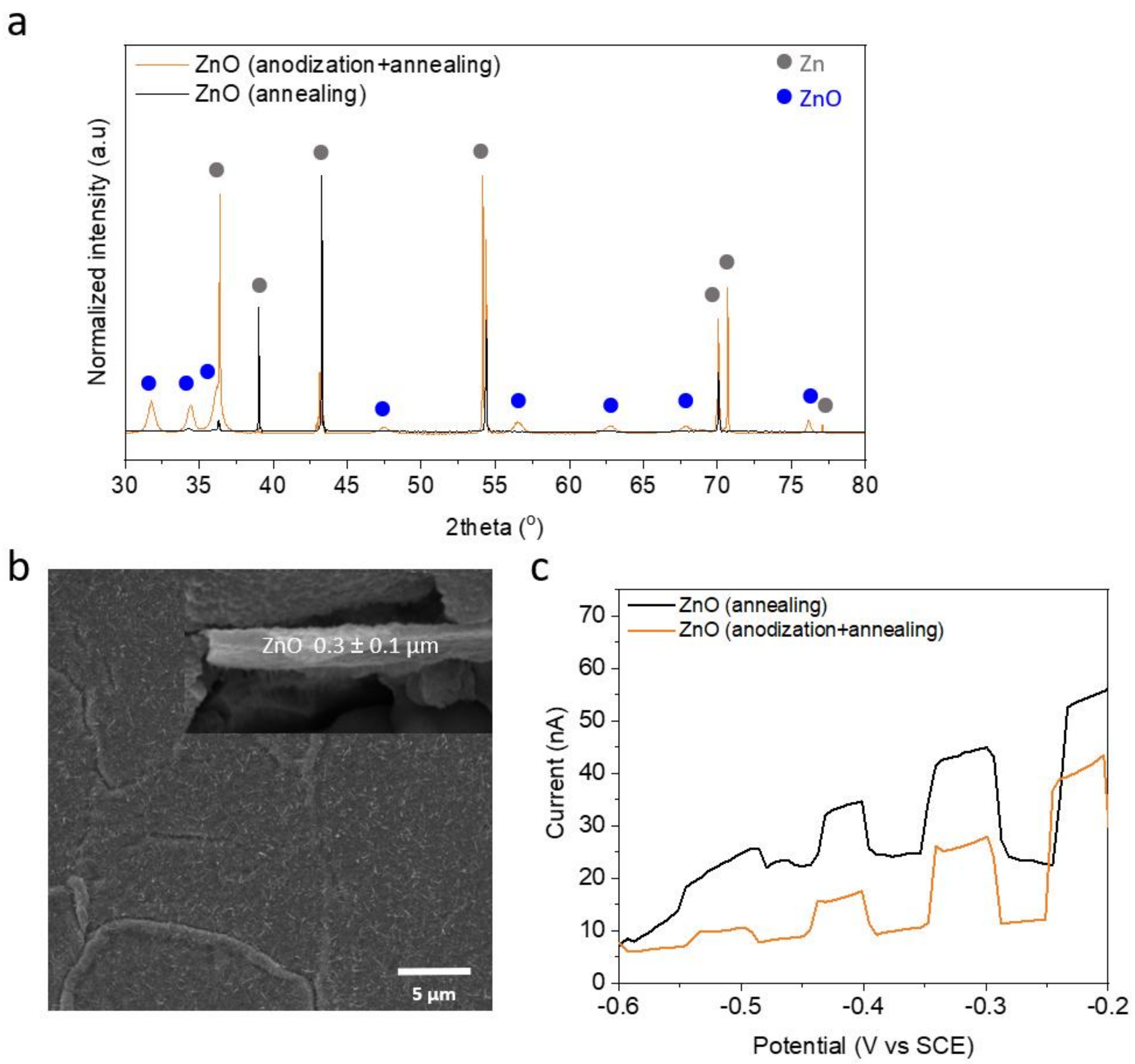

Figure S7. (a) XRD diffraction patterns of a ZnO/Zn foil obtained by direct annealing (black) and anodization+annealing (orange). (b) SEM images of a ZnO/Zn foil obtained by direct annealing. The insets present the thickness of the ZnO film. (c) LSVs recorded with ZnO/Zn/phosphate fibers obtained by annealing (black) and anodization+annealing (orange) under intermittent illumination with λ_{exc} = 340 nm in 2 M K-borate solution (pH 9.5). Scan rate = 10 mV·s^{-1}.

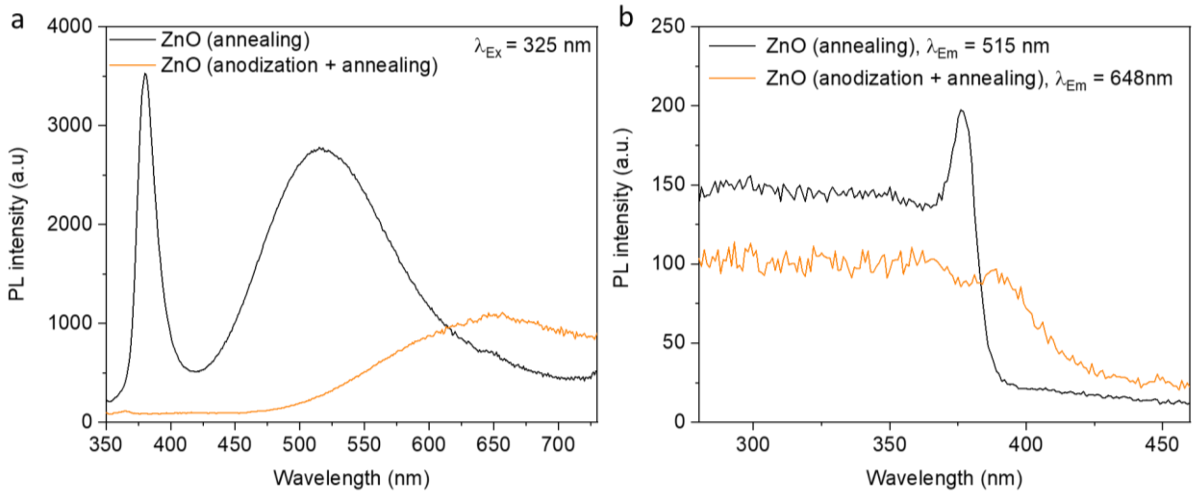

Figure S8. Photoluminescence (PL) spectra measured on ZnO/Zn foils after direct annealing (black) and after anodization+annealing (orange). (a) PL emission spectra measured with an excitation of 325 nm and (b) PL excitation spectra at an emission of 515 and 648 nm.

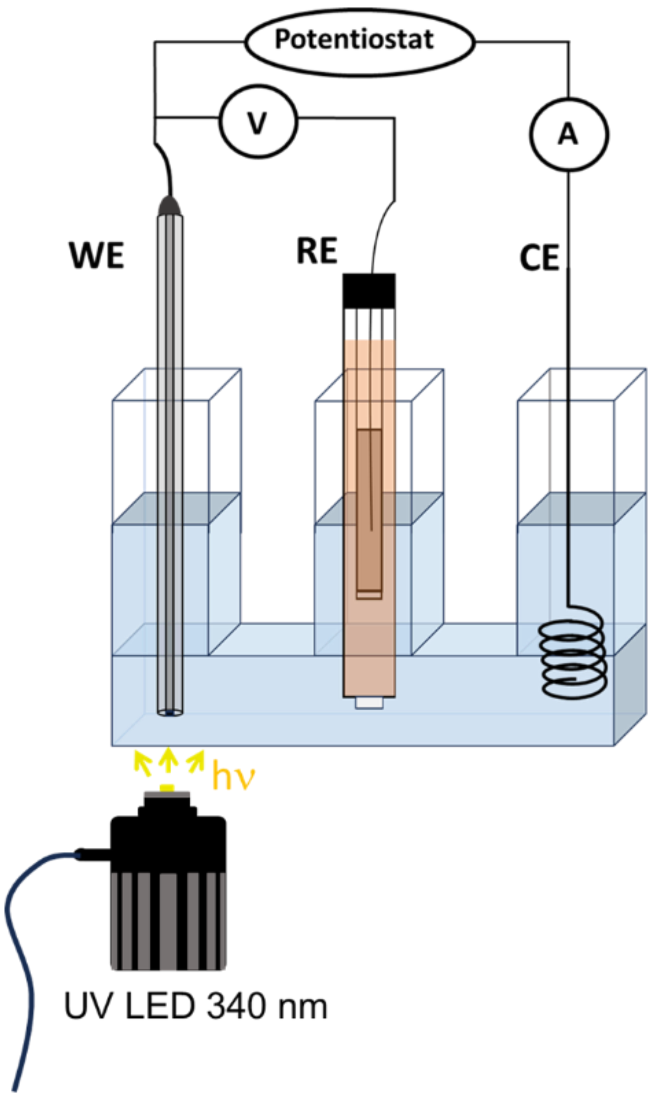

Figure S9. Scheme of the photoelectrochemical set-up.

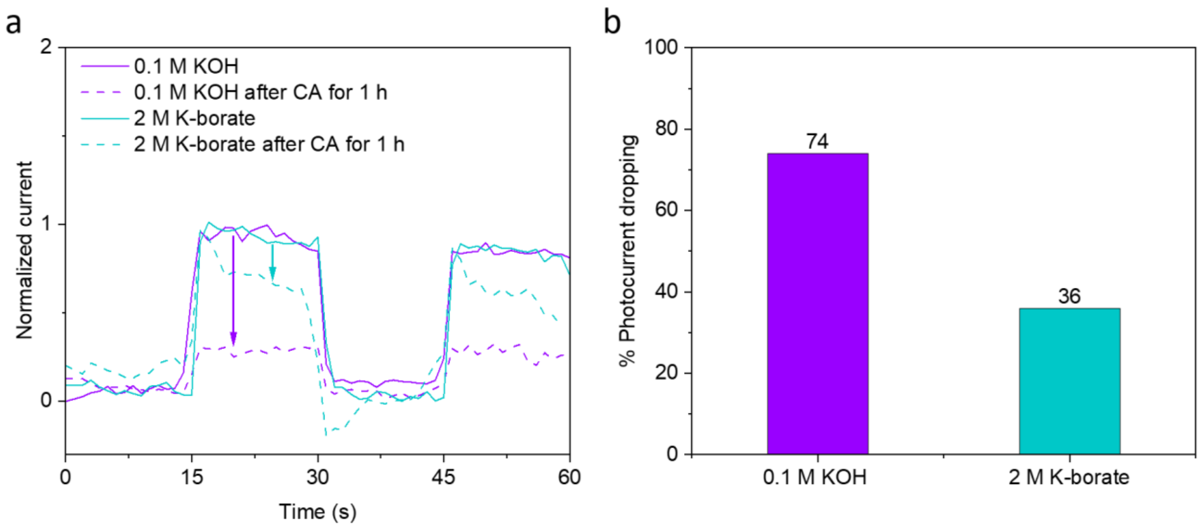

Figure S10. (a) Normalized chronoamperograms recorded at -0.3 V under intermittent illumination (illumination frequency = 33 mHz) in 2 M K-borate (blue) and 0.1 M KOH (purple) before and after chronoamperometry (CA) under illumination for 1 h. (b) Histogram showing the percentage of photocurrent loss after CA recorded at -0.3 V for 1 h with a ZnO/Zn/phosphate optrode in 2 M K-borate (purple) and 0.1 M KOH (blue). Photocurrent dropping was calculated from the photocurrent before (i_{before}) and after (i_{after}) CA under 1 h illumination. %Photocurrent dropping = (i_{before} - i_{after}) × 100 / i_{before}.

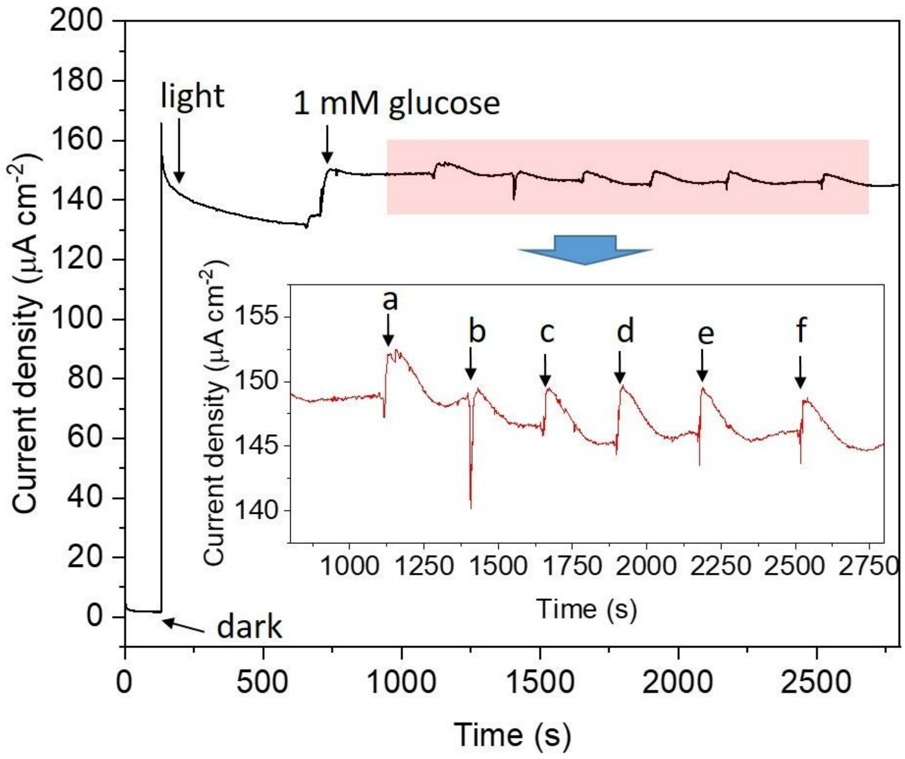

Figure S11. Interference ability test performed on a ZnO/Zn foil photoanode. Amperometric response under LED light (340 nm) illumination of the ZnO on Zn foil electrode with the addition of 1 mM glucose and 0.2 mM of each possible interfering species i.e. (a) ascorbic acid, (b) dopamine, (c) NaCl, (d) $MgCl_2$, (e) $CaCl_2$, and (f) L-cysteine in the 2 M K-borate (pH 9.5) solution at +0.16 V (versus SCE).

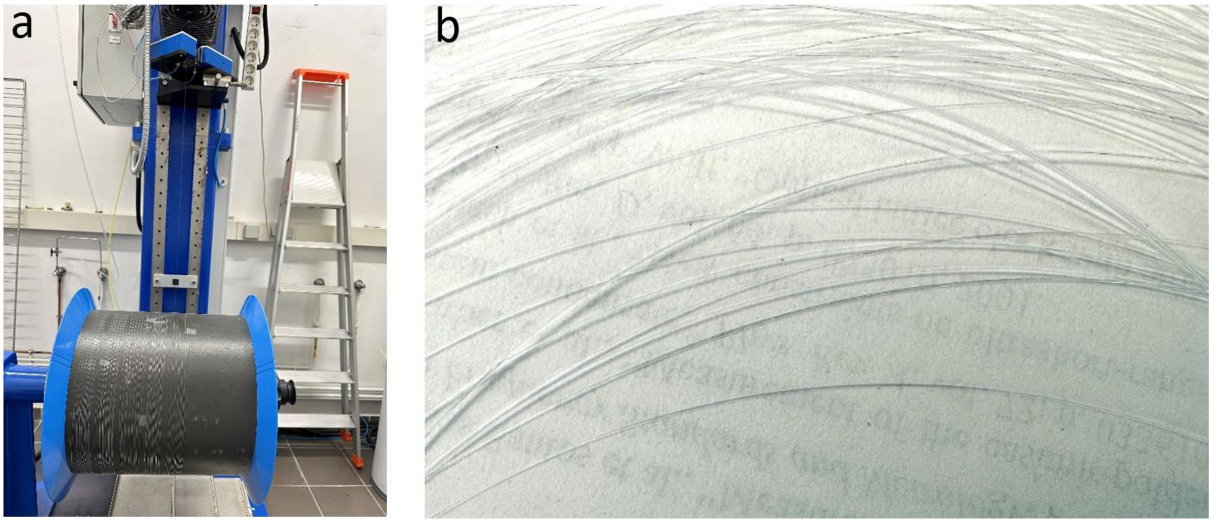

Figure S12. Photographs showing a) the several-meter-long multimaterial fiber wrapped around the “drum” during drawing. b) Several multimaterial fibers that are bent.

Table S1. Comparison of the performance of ZnO-based sensors reported for the detection of glucose.

**Nanomaterials**	**Technique** [a]	**Sensitivity ($\mu A \cdot cm^{-2} \cdot mM^{-1}$)**	**Linear range (mM)**	**Refs.**
ZnO	PEC	18.74	1-10	This work
ZnO@SS NRs	PEC	0.76	0.01-0.1	[1]
MWCNT/ZnO QDs	PEC	9.36	0.0001-0.0025	[2]
Cu/ZnO Nano-Thorn	PEC	63.76	0.1-4.5	[3]
ERGO/ZnONWs/CdS	PEC	430.06	0.01-1	[4]
		25.75	1-15	
ZnO/CNO	EC	606.64	0.1-15	[5]
ZnO/MWCNT/GCE	EC	64.29	1-10	[6]
ZnO/MXene	EC	29	0.05–0.7	[7]

[a]: PEC : photoelectrochemical, EC : electrochemical

References

1. W. Liu, W. Zhan, X. Jia, Q. Liu, R. Chen, D. Li, Y. Huang, G. Zhang, H. Ni, Rapid synthesis of vertically-aligned zinc oxide nanorods on stainless steel for non-enzymatic glucose and H_2O_2 photoelectrochemical sensor, *Appl. Surf. Sci.* 480, (2019): 341, https://doi.org/10.1016/j.apsusc.2019.01.236

2. V. Vinoth, G. Subramaniyam, S. Anandan, H. Valdés, and P. Manidurai, "Non-enzymatic glucose sensor and photocurrent performance of zinc oxide quantum dots supported multi-walled carbon nanotubes", *Mater. Sci. Eng. B* 265, (2021): 115036,

https://doi.org/10.1016/j.mseb.2020.115036

3. B. Yang, N. Han, S. Hu, *et al.*, "Cu/ZnO nano-thorn with modifiable morphology for photoelectrochemical detection of glucose", *J. Electrochem. Soc.* 168, (2021): 027516,

https://doi.org/10.1149/1945-7111/abe50e

4. E. P. Gür, Z. S. B. Yılmaz, S. Dinç, and Ü. Demir, "A self-powered photoelectrochemical and non-enzymatic glucose sensor based on ERGO/ZnONWs/CdS photoanode", *Electrochim. Acta* 501, (2024): 144825,

https://doi.org/10.1016/j.electacta.2024.144825

5. A. Sharma, A. Agrawal, G. Pandey, S. Kumar, K. Awasthi, and A. Awasthi, "Carbon nano-onion-decorated ZnO composite-based enzyme-less electrochemical biosensing approach for glucose", *ACS Omega* 7, no. 42 (2022): 37748,

https://doi.org/10.1021/acsomega.2c04730

6. A. Tarlani, M. Fallah, B. Lotfi, *et al.*,"New ZnO nanostructures as non-enzymatic glucose biosensors", *Biosens. Bioelectron.* 67, (2015): 601,

https://doi.org/10.1016/j.bios.2014.09.063

7. V. Myndrul, E. Coy, N. Babayevska, *et al.*, "MXene nanoflakes decorating ZnO tetrapods for enhanced performance of skin-attachable stretchable enzymatic electrochemical glucose sensor", *Biosens. Bioelectron.* 207, (2022):114141,

https://doi.org/10.1016/j.bios.2022.114141
